# Application of regularized Richardson–Lucy algorithm for deconvolution of confocal microscopy images

**DOI:** 10.1111/j.1365-2818.2011.03486.x

**Published:** 2011-08

**Authors:** M Laasmaa, M Vendelin, P Peterson

**Affiliations:** Laboratory of Systems Biology, Institute of Cybernetics, Tallinn University of TechnologyTallinn, Estonia

**Keywords:** Deconvolution, estimating regularization parameters, fluorescence microscopy, 3D quantitative fluorescence microscopy imaging

## Abstract

Although confocal microscopes have considerably smaller contribution of out-of-focus light than widefield microscopes, the confocal images can still be enhanced mathematically if the optical and data acquisition effects are accounted for. For that, several deconvolution algorithms have been proposed. As a practical solution, maximum-likelihood algorithms with regularization have been used. However, the choice of regularization parameters is often unknown although it has considerable effect on the result of deconvolution process. The aims of this work were: to find good estimates of deconvolution parameters; and to develop an open source software package that would allow testing different deconvolution algorithms and that would be easy to use in practice. Here, Richardson–Lucy algorithm has been implemented together with the total variation regularization in an open source software package IOCBio Microscope. The influence of total variation regularization on deconvolution process is determined by one parameter. We derived a formula to estimate this regularization parameter automatically from the images as the algorithm progresses. To assess the effectiveness of this algorithm, synthetic images were composed on the basis of confocal images of rat cardiomyocytes. From the analysis of deconvolved results, we have determined under which conditions our estimation of total variation regularization parameter gives good results. The estimated total variation regularization parameter can be monitored during deconvolution process and used as a stopping criterion. An inverse relation between the optimal regularization parameter and the peak signal-to-noise ratio of an image is shown. Finally, we demonstrate the use of the developed software by deconvolving images of rat cardiomyocytes with stained mitochondria and sarcolemma obtained by confocal and widefield microscopes.

## Introduction

In biosciences, fluorescence microscopy is an extremely useful and important method for studying living organisms. As one of the implementations of fluorescence microscopy, confocal fluorescence microscopy can be used to study live cells and analyse the response of the cells to external stimuli. Confocal microscopy has several advantages over traditional widefield microscopy. The main advantage is the ability to produce in-focus images of thick specimens via elimination or reduction of background information outside of the focal plane and ability to control the depth of field (within the accuracy of an Airy disk size) (Inoué, 2006). Despite the advantages over widefield microscopy, confocal images contain imperfections, for example, aberrations due to nonideal optical pathway, residual out-of-focus light, noise from detector electronics, etc. (Shaw, 2006).

In this paper we focus on image enhancement of microscope images by deconvolution (Cannell *et al.*, 2006). Each microscope alters the appearance of specimens in a specific way. Image formation can be described by the mathematical operation of convolution, where the ‘true’ image is convolved with distortion effects from the microscope. Deconvolution is a method to reverse the aberrations caused by convolution, that is remove the distortions of the optical train, contributions from out-of-focus objects, and with regularization enabled, reduce the noise originated from detector electronics. Deconvolution takes into account microscope optics and the nature of noise. Therefore, it is a method that can efficiently enhance both widefield microscopy and confocal microscopy images. It can considerably improve image contrast and reduce noise in microscope images.

Several deconvolution algorithms have been proposed for three-dimensional (3D) microscopy. For example, noniterative algorithms such as regularized inverse-filtering algorithm (Preza *et al.*, 1992), nearest-neighbour algorithm, Wiener filtering algorithm (Shaw & Rawlins, 1991a), etc.; iterative algorithms such as Richardson–Lucy (RL) algorithm (Richardson, 1972; Lucy, 1974), Jansson-van Cittert algorithm (Agard, 1984; Abdelhak & Sedki, 1992), Carrington algorithm (Carrington *et al.*, 1995), constrained Tikhonov-Miller algorithm (van Kempen *et al.*, 1997), Fourier-wavelet regularized algorithm (Neelamani *et al.*, 2004), expectation maximization algorithm (Conchello, 1998; Preza & Conchello, 2004), etc; blind deconvolution algorithms (Holmes, 1992; Avinash, 1996; Markham & Conchello, 1999). Usually, noniterative methods are fastest but these do not provide optimal image quality, especially in the presence of noise (Cannell *et al.*, 2006). The particular choice of deconvolution algorithm depends on users requirements (should the deconvolved image be pleasant to the viewers eye or be quantitatively as correct as possible), computational resources and limitations (Cannell *et al.*, 2006; Sun *et al.*, 2009).

In this paper, we analyse the RL iterative algorithm that is derived for Poisson noise (Richardson, 1972; Lucy, 1974). The assumption of Poisson noise is adequate for confocal microscopes because these use photodetection devices such as avalanche photodiodes to count the number of photons that are emitted from specimens. Because of the quantum nature of light, the number of detected photons is a Poisson process whose variance is equal to the mean of counted photons.

The RL algorithm is commonly used for telescope and microscope image enhancement (Dey *et al.*, 2006). An undesired property of the RL algorithm is that, in the presence of noise, the deconvolution process converges to a solution which is dominated by the noise (Dey *et al.*, 2004). An option to circumvent this, is to prefilter images (Cannell *et al.*, 2006). Another option is to introduce regularization terms such as Tikhonov–Miller (van Kempen & van Vliet, 2000) or maximum entropy to the RL algorithm (de Monvel *et al.*, 2001, 2003). Algorithms which are based on Tikhonov–Miller regularization, are often used for deconvolving 3D images. Such algorithms avoid noise amplification but operate poorly near the object edges. Alternatively, to increase the sharpness of object borders and obtain smooth homogeneous areas, total variation (TV) regularization is often applied in the RL algorithm (Dey *et al.*, 2004). However, regularization terms contain unknown parameters that must be carefully chosen to achieve optimal deconvolution results that would be as close as possible to the ‘true’ image. Some regularized algorithms provide means to determine how much regularization to use in each restoration step (Sun *et al.*, 2009; Liao *et al.*, 2009). In this paper, we introduce a method to estimate the regularization parameter for the regularized RL deconvolution algorithm.

All iterative deconvolution algorithms have to deal with the problem of stopping the iteration process. Provided that the iteration converges, seemingly the most natural, in fact, also the most popular stopping criteria are based on measuring the stationary state of the iteration process. For example, this can be measured by computing the relative changes of subsequent estimates and specifying a stopping threshold (Dey *et al.*, 2004, 2006; Sun *et al.*, 2009). Surprisingly, as we show in this work, such stopping criteria turn out to be suboptimal: the converged estimate may be less accurate (when comparing with the ‘true’ image) than some of the intermediate estimates. So, a better stopping criteria is needed for improving quantitative results of iterative deconvolution algorithms.

For image restoration by deconvolution, both commercial and open source computer programs are available. Commercial image restoration software solutions give good results in image enhancement and are easy to use, but, as a drawback, they are expensive and do not support testing alternative deconvolution algorithms due to their closed source development policy. Several open source software libraries exists that implement various deconvolution algorithms (Peterson, 2010b). For example, Clarity Deconvolution Library (Quammen, 2007) (GPL license) is a C/C++ library that currently implements Wiener filtering (Shaw & Rawlins, 1991b), Jansson-van Cittert iterative (Agard, 1984), maximum likelihood iterative (Richardson, 1972; Lucy, 1974) with symmetric point spread function (PSF) algorithms; COSMOS (Valdimarsson & Preza, 2007) is a C++ library (GPL, the successor of XCOSM software) that currently implements depth variant expectation maximization (Preza & Conchello, 2004), a linear least square (Preza *et al.*, 1992), a linear maximum *a posteriori* (Preza *et al.*, 1993), the Jansen-van Cittert (Agard, 1984) and the expectation maximization (Conchello, 1998) algorithms; Deconv is a C++ library (GPL) that currently implements maximum likelihood-Landweber, -conjugate gradient and -expectation maximization iterative deconvolution (Sun *et al.*, 2009) algorithms. For a scientist who prefers to focus on solving scientific problems, this variety of software and algorithms makes it difficult to decide which of the algorithms is most suitable for particular image data and available computational resources. Therefore, a software platform is needed that would support testing and comparing different deconvolution algorithms and their implementations in an unified manner for variety of microscopy image file formats. For this, we use Python programming language that is becoming an increasingly popular choice for scientific computing because of its many features that are attractive for scientists: Python has very clean and easy-to-learn syntax, it supports very high-level object-oriented programming paradigm, and is easy to extend. High-quality scientific computational packages in Python have emerged within the last 10 years (Oliphant, 2007; Jones *et al.*, 2001) and well-developed tools exist for interfacing existing C/C++ and Fortran libraries to Python (Beazley, 2003; Peterson, 2009).

The aims of this work are: (1) to work out a practical method for using deconvolution algorithms, in particularly, to find good estimates to regularization parameters as well as to establish a robust criterion for stopping iteration process that would give closest result to the ‘true’ image rather than just detecting deconvolution process stationarity; (2) to develop an open source software package that would allow testing different deconvolution algorithms and at the same time would be easy to use in practice.

## Material and methods

### Description of the deconvolution process

To deconvolve microscope images we use the RL algorithm (Richardson, 1972; Lucy, 1974). The algorithm is based on the following mathematical image formation model:

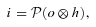
(1)where *i* represents the recorded image stack represented as 3D array, where each item value corresponds to the intensity of a measured voxel, *o* is the object, *h* is the PSF defined by the optical train of a specific microscope, ⊗ denotes convolution operation, 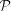 represents Poisson noise originating from counting photons. With maximum likelihood approach and TV regularization, the model provides the following equation (Dey *et al.*, 2004):

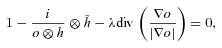
(2)where 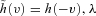 is regularization parameter and differentiation operations are defined with respect to voxel coordinates *v*. From Eq. (2), a multiplicative gradient-type RL algorithm for one iteration can be derived (Dey *et al.*, 2004):

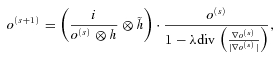
(3)whereby in this paper we use *o*^(0)^=*i*. In general, the initial estimate *o*^(0)^ can be denoised, for example, by convolving *i* with *h*, or applying Gaussian filter to *i*, etc. Note also that Eq. (3) may introduce negative values to deconvolution estimate. This happens when the denominator of Eq. (3) becomes negative for some voxel value. The negativity usually indicates unstable deconvolution process due to an inappropriate choice of λ value. In such cases, the iteration process should be stopped immediately.

We denote the result of deconvolution with the above scheme as *o*^(*S*)^=*i*⊗^−1^_λ,*S*_*h*, where *S* denotes the number of iteration steps.

### Estimation of the TV regularization parameter value

Let us define a functional
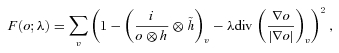
(4)which ought to have zero value when Eq. (2) is fulfilled; *v*= (*i*, *j*, *k*) defines the location of a voxel in 3D image. At the *s*th deconvolution iteration, the regularization parameter λ can be chosen such that *F*(*o*^(*s*)^; λ) is minimal. It is easy to show that the minimal value of the functional Eq. (4) is *F*(*o*^(*s*)^; λ^(*s*)^_lsq_) where
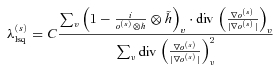
(5)and formally *C*= 1. The coefficient *C* is chosen such that at the first iteration the λ value is close to the optimal λ value (see Results), that is, λ^(0)^_lsq_≡ 50/*SNR* where *SNR* denotes peak signal-to-noise ratio (SNR) of the recorded image *i*[Eq. (6)].

Note that λ_lsq_ in Eq. (5) is closely related to Lagrange multiplier method used in (Gilboa *et al.*, 2003) when taking 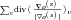 as a constant that describes texture variations in the estimate *o*^(*s*)^.

### Estimation of the peak SNR

To quantify the noise level in recorded images, we use the peak SNR. The peak SNR is defined as the ratio of mean to standard deviation of the brightest part of the recorded images. Because all of our image data is recorded with a photon counting module then the peak SNR can be directly estimated from Poisson statistics: it is the square of mean photon count in the brightest part of an image. The mean photon count is estimated as a maximum value of an averaged image. The averaging of the image is carried out with 3 × 3 × 3 uniform kernel.

In summary, the SNR of a recorded image *i* that values are counts of detected photons per voxel time, is
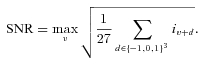
(6)

### Numerical methods

The deconvolution algorithm is implemented in Python programming language (van Rossum, 1991) and released as open source software IOCBio Microscope (Peterson, 2010a). For array operations, the NumPy package is used. For correcting PSF to correct voxel size, the SciPy (Jones *et al.*, 2001) Ndimage package is used. Convolution operation is carried out via FFT using FFTW library (Frigo & Johnson, 2005) and the numerical scheme for computing 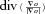 is implemented in C programming language for better performance. The original scheme for computing 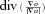 as given in (Dey *et al.*, 2004) has a typo and below follows the corrected scheme:

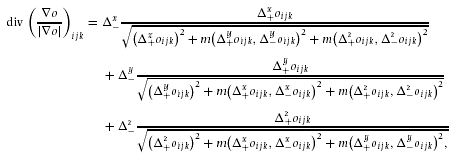
(7)where
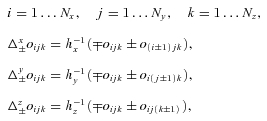
(8)
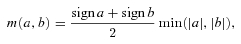
(9)and *h_x_*, *h_y_*, *h*_*z*_ are voxel dimensions. In boundary points, the following relations are used (Dey *et al.*, 2004):

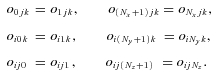
(10)

### Determination of the PSF

The accuracy of any deconvolution algorithm depends on the quality of used PSF. The PSF can be computed from the optical properties of a microscope system or estimated from the measurements of microspheres. Also, a third option exists where the PSF is estimated from recorded images together with observed objects (blind deconvolution) but for this paper we assume that PSF is known before executing the deconvolution process.

From the deconvolution quality point of view the estimation of PSF is preferred over the computed PSF because all optical aberrations of the given microscope system are taken into account. By contrast, in estimating PSF from microspheres measurements, the problem of suppressing noise must be tackled. In Lai *et al.* (2005), a PSF denoising method is introduced that is based on singular value decomposition. The method has disadvantage that it produces small but visible artificial ripples to the denoised PSF.

In this paper, the PSF is estimated from the microscope images of fluorescent microspheres using the following algorithm:

1. Determine the location of microspheres and extract their intensity profiles.
2. Sum the intensity profiles, to form the PSF function *h*. The SNR ratio will increase with the increase of the number (*M*) of intensity profiles.

It turns out that further denoising procedures on the summed PSF function is not required when *M* is sufficiently large: the SNR of a single PSF measurement increases approximately 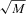 times when summing up *M* different PSF measurements. In our PSF cases, typical values for *M* are within range 4–12.

For this paper, two PSFs for a confocal microscope are estimated for laser lines 473 and 633 nm. For laser line 473 nm, we used microspheres (green) with excitation maximum at 505 nm and emission maximum at 540 nm. Emission was collected through a bandpass filter 550 ± 44 nm (FF01-550/88-25, Semrock). For laser line 633 nm, we used microspheres (deep red) with excitation maximum at 633 nm and emission maximum at 660 nm. Emission was collected through a bandpass filter 725 ± 75 nm (FF01-725/150-25, Semrock).

In addition, a PSF for a widefield microscope was obtained by exciting microspheres (orange – excitation maximum at 540, emission maximum at 560 nm) with fluorescent light through a bandpass filter 543 ± 22 nm (Semrock, Rochester, NY, U.S.A.) and emission was collected through a bandpass filter 593 ± 40 nm (Semrock).

**Fig. 1:**
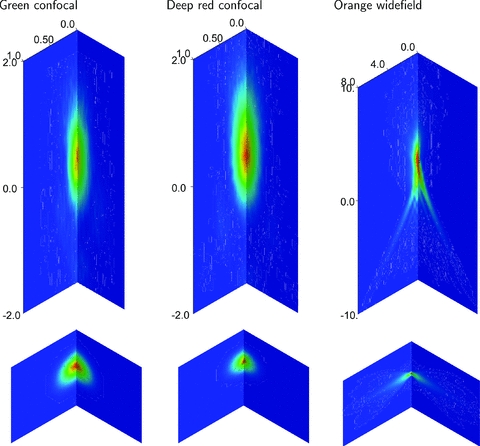
The PSFs of the confocal and the widefield microscope used in this study. The upper row shows three PSFs estimated from the measurements of microspheres; two confocal PSFs and a widefield PSF. The axis in the subplots show distance in μm; note the difference in scales used for confocal and widefield PSFs. Voxel sizes for PSF were as follows: green confocal 0.019 × 0.019 × 0.138 μm, deep red confocal 0.027 × 0.027 × 0.120 μm and orange widefield 0.132 × 0.132 × 0.276 μm, respectively. The lower row shows the corresponding optical transfer functions. Note that the PSF of widefield microscope is larger than the PSFs for confocal microscope.

PSFs that were used in this study are shown in Fig. 1 in the upper row. The lower row in Fig. 1 shows the corresponding optical transfer functions for all PSFs.

All types of microspheres have diameter 0.175 μm (PS-Speck, Molecular Probes, Invitrogen, Eugene, OR, U.S.A.).

The slides of microspheres for measuring PSFs were prepared as follows. A 1000-fold dilution in water was made from the original suspension. A small drop of the dilution was placed on a cover glass of 0.17 mm in thickness and let it dry in air. When the sample was dry a small drop of immersion oil with refractive index 1.334 at 23° (Carl Zeiss Immersol™ W, Oberkochen, Germany) was added on the spot and fixed with a glass slide.

Imaging of confocal images was carried out with custom confocal laser scanning microscope with a digital photon counter (avalanche photodiode, Perkin Elmer, SPCM-AQRH-13, Vaudreuil, Canada) and 60× water-immersion objective (Olympus, UPLSAPO 60XW/1.2, Hamburg, Germany). Imaging of widefield images was carried out with Nikon TiU microscope (Nikon, Amstelveen, the Netherlands), equipped with Andor EMCCD camera (iXon 885, Andor, Belfast, Ireland), 60× water-immersion objective (Nikon, Plan Apo VC 60×/1.2 WI, Amstelveen, the Netherlands). Optical sectioning was carried out by piezoelectric objective positioning system (Piezosystem Jena GmbH, MIPOS 250SG M25, Jena, Germany).

### Obtaining experimental data for examples

Rat cardiomyocytes were isolated as in Sepp *et al.* (2010). Live cells were imaged using the approach similar to Birkedal *et al.* (2006) and Vendelin & Birkedal (2008). In short, the cells were kept and imaged in solution consisting of (mM): KH_2_PO_4_ 3, MgCl_2_ 3, sucrose 110, K-lactobionate 60, taurine 20, HEPES 20, EGTA 0.5, DTT 0.5, malate 2, glutamate 5 and 5 mg mL^−1^ BSA. pH was adjusted to 7.1 with KOH at 25°. Mitochondria were visualized by staining isolated cells with MitoTracker Green FM with the final concentration of 200 nm; for sarcolemma we used di-8-ANEPPS with the final concentration of 1 μM (both from Invitrogen). After incubation for 15 min at the room temperature, cells were washed and inserted to imaging chamber that consisted of a FlexiPERM silicone insert (Vivascience, Hanau, Germany) attached to a cover slip glass of 0.17 mm in thickness.

For acquisition of images, the same microscope set-ups were used as described earlier for measuring PSF.

### Analysis of the deconvolution process

To study the effects of deconvolution, we created two types of synthetic images with different textures from the microscope images of mitochondria and sarcolemma of rat cardiomyocytes. The image of mitochondria gives a typical example of blocky like textures. By contrast, the image of sarcolemma gives a typical example of honeycomb-like textures. The synthetic images were obtained from confocal microscope images as follows. A microscope image was convolved and deconvolved several times using the following algorithm:

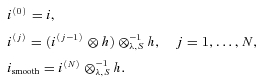
(11)Note that the number of deconvolutions is larger by 1 compared to the number of convolutions in Eq. (11) to obtain small details in *i*_smooth_ that would be of similar size to the small details in the object image *o*[Eq. (1)]. Our smooth synthetic images were obtained using the following parameters: *N*= 4, *S*= 200, λ= 0.

**Fig. 2:**
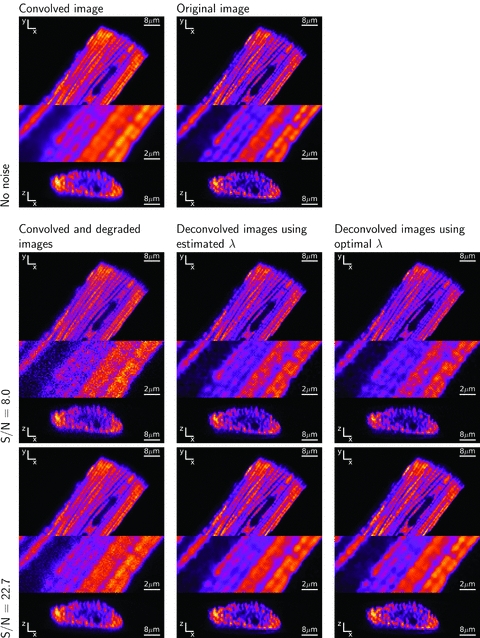
The results of deconvolving synthetic 3D images with the voxel size 0.136 μm × 0.136 μm × 0.707 μm. The first row shows convolved and original images, respectively. The original synthetic image is obtained from the confocal image of mitochondria in cardiac cell and has typical blocky like texture. The first column shows degraded images with different signal-to-noise ratios (SNR); the second column represents degraded images that are deconvolved using estimated λ; and the last column shows degraded images that are deconvolved using an optimal value for λ. Test images with different SNR and the deconvolution results are shown in rows, starting from second, in decreasing order according to SNR. Deconvolution results correspond to minimal MSE value.

We prefer using such synthetic images over traditional artificial images, which represent various geometrical shapes, because synthetic images allow us to tune the deconvolution algorithm parameters for microscope images that biologists need to deconvolve. An example of a synthetic image with blocky-like texture is shown in the first row of the second column of Fig. 2.

Two sets of test images were generated by convolving the synthetic image with PSF and degrading with Poisson noise. Various SNRs (13 different SNR values in total) were obtained by scaling the values of the synthetic image before degrading. For example of degraded image, see the first column of Fig. 2. The sets of test images were deconvolved using different regularization parameter λ values (100 different λ values in total) and compared with the synthetic image.

To quantify the quality of the deconvolution, we use mean squared error (MSE) between original object (e.g. synthetic image) and deconvolved images
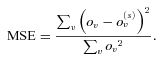
(12)

In addition, we follow deconvolution process by computing relative changes between two estimates
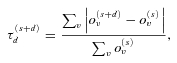
(13)where *d* shows the distance between the two estimates in iteration steps. In this paper, we consider only τ_1_ and τ_2_.

## Results

The RL deconvolution algorithm with TV regularization has been shown to give good deconvolution results with a carefully chosen regularization parameter λ. Because finding good parameter value to the algorithm is difficult, our aim is to estimate this value from microscope images. To test the effectiveness of the deconvolution method, we deconvolved synthetic images that were degraded with various Poisson noise levels. Following this, deconvolution of images acquired on confocal and widefield microscopes are shown to demonstrate the effectiveness of the algorithm in practice.

### Deconvolving synthetic images

The effectiveness of the deconvolution method can be assessed with the use of a synthetic image which is convolved and degraded with different Poisson noise levels. To ensure that this synthetic image represents similar textures as in cells, we used images of mitochondria and sarcolemma in rat cardiomyocyte where the noise was smoothed out by robustly convolving and deconvolving the image several times [Eq. (11)].

For example, the synthetic image from mitochondria recording is presented in the first row of the second column of Fig. 2. The blocky-like texture in this synthetic image consists of different geometrical shapes and intensities: spherical shapes, lines, homogeneous and heterogeneous areas. Two test images with different SNRs are shown in the first column of Fig. 2. The second and third columns of Fig. 2 show images deconvolved using our λ estimation procedure [Eq. (5)] and optimal regularization parameter λ_opt_, respectively. The procedure for determining λ_opt_ is described later. Because for synthetic images the original image is known, the actual efficiency of the deconvolution algorithm can be assessed directly by using the MSE [Eq. (12)]. The MSE provides a mean to measure the difference between the deconvolved and original image. The larger MSE value corresponds to larger difference between images.

**Fig. 3:**
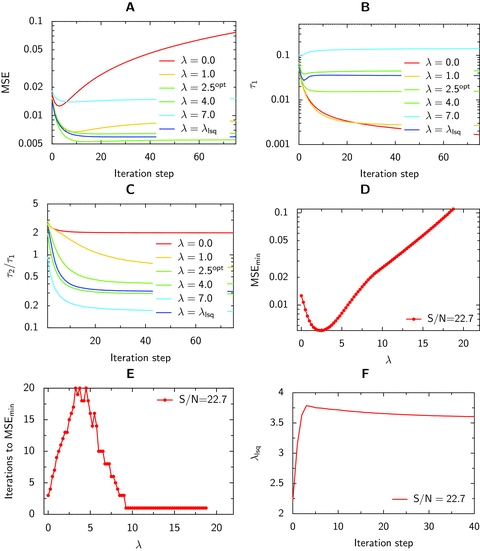
The analysis of deconvolution for degraded image of blocky-like texture with Poisson noise, SNR = 22.7. (A) The evolution of the mean squared error (MSE) during iteration. The deconvolution process converges to a solution that is slightly different from the original image: MSE stabilizes to a certain nonzero level. The nearest result to original image is achieved with λ= 2.5 at iteration step 15 when MSE is minimal. (B) The evolution of relative change between estimates. Here τ_1_ denotes the change between iterations *s* and *s*+ 1. Note that the decrease of τ_1_ is in correlation with the convergence of MSE whereas smaller τ_1_ does not mean smaller MSE. (C) The evolution of the ratio between two relative changes. Symbol τ_2_ denotes the relative change between iterations *s* and *s*+ 2. (D) Minimal MSE as a function of λ. The minimum point of the graph defines optimal λ value for that case. (E) The number of iteration steps required to achieve minimal MSE for different λ values. (F) The evolution of estimated λ during iteration. Note that estimated λ obtains its maximum at third iteration.

**Fig. 4:**
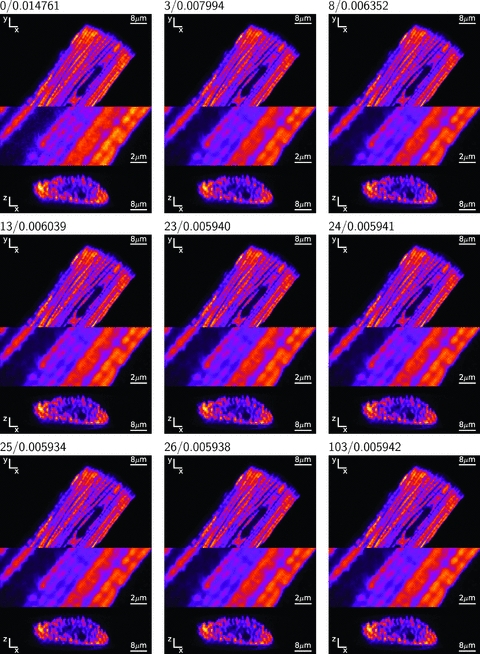
The results of deconvolving synthetic 3D images with SNR = 22.7. The numbers above images show the iteration step and MSE between original object and deconvolved image. On the iteration step *s*= 3, the λ_lsq_ has maximal value and on the iteration step *s*= 25 the MSE has minimal value for this specific case. Note that there is no visible differences between results from the iteration step *s*= 25 and *s*≥ 3.

Fig. 3A shows the MSE between the original and estimated image for each iteration of the deconvolution process. The MSE was computed from the results which were obtained by deconvolving degraded original image with Poisson noise such that SNR = 22.7 using the λ estimation formula Eq. (5) and various fixed λ values. In the presence of noise, we see that the MSE is smaller when using the RL algorithm with TV regularization rather than the traditional RL algorithm (λ= 0). Looking at Fig. 3A, we see that when λ is small, the MSE between the original and deconvolved images decreases and reaches its minimum in a small number of iterations, after which the MSE starts to increase monotonically. When the regularization parameter value is larger (e.g. λ= 4.0), the deconvolution process stabilizes shortly after passing the MSE minimum. However, at larger λ values (λ= 7.0), the deconvolution process is not able to enhance image considerably which is clear from observation of MSE. Although deconvolving the test image with SNR = 22.7, the lowest MSE = 0.00528 was obtained at the 15th iteration step with regularization parameter λ= 2.5. When using our estimated λ, the deconvolution process reached its best result at the 25th iteration step with MSE = 0.000593. The results of 0th, 3rd, 8th, …, 103th, iterations are given in Fig. 4. Note that λ_lsq_ achieves its maximum at third iteration and MSE achieves its minimum at 25th iteration. Our stopping criterion suggests stopping iteration at 8th step when MSE = 0.00635 for this particular case.

The behaviour of the deconvolution process was tracked using the relative change between two successive estimates [Eq. (13)], which is shown in Fig. 3B. Frequently, the relative change between two successive estimates τ_1_ is used as a stopping criterion (Cannell *et al.*, 2006; Dey *et al.*, 2006). According to that criterion, if τ_1_ falls down to a given threshold the deconvolution process is stopped. However, there are at least two problems when using this criterion in practice, as indicated later.

First notice that, ideally, the stopping criterion should finish the deconvolution process when the deconvolved image is closest to ‘true’ image. From the analysis of MSE and τ_1_ evolutions, it is clear that this is not always true (Figs 3A and B). Namely, as it is shown in the figure, there is no τ_1_ value that can be used as an universal threshold for all regularization parameter values. Although it is theoretically possible to choose a threshold value for each trace individually so that deconvolution will be stopped at minimum of MSE, finding such threshold value in practice is very difficult, if impossible.

Secondly, from the comparison of deconvolution processes with different TV regularization parameter values reveals that the convergence of deconvolution is not always related to small τ_1_ values. Namely, lower τ_1_ value would indicate stabilization of the deconvolution process and we would expect the MSE to stabilize as well. Comparison of Figs 3A and B at λ= 1.0 shows that the relative change τ_1_ is monotonically decreasing but MSE starts to increase after the sixth iteration. In contrast to that, at λ= 2.5, the relative change τ_1_ has higher values than at λ= 1.0, but from the trace of the MSE (Fig. 3A), the deconvolution process seems to be converged because the MSE remains relatively constant at λ= 2.5. So, there exists no such threshold value for τ_1_ that would be applicable for both cases.

It turns out that the behaviour where τ_1_ is relatively large and MSE is stationary, indicates oscillations between several successive iteration steps. To demonstrate that, let us define the ratio between two different relative changes τ_2_/τ_1_, where τ_1_ denotes the relative change between *s* and *s*+ 1 and τ_2_ denotes the change between *s* and *s*+ 2 iteration steps. Following the evolution of τ_2_/τ_1_ in Fig. 3C at λ= 2.5, the ratio decreases and stabilizes to a level smaller than 1. By contrast, using λ= 0 (Fig. 3A), the ratio stabilizes at a level above 1 while the MSE increases during iteration. Thus, for the cases where the ratio falls under 1 and stabilizes, we can assume that the deconvolution process starts to oscillate between several successive iteration steps. However, for the case where τ_2_/τ_1_ is larger than 1, we can assume that the changes in the images are progressive during deconvolution leading to changes in the MSE, as for λ= 0.

### Optimal TV regularization parameter value and stopping criterion

As it is shown in Fig. 3A, the minimal MSE value is different for each λ value. Fig. 3D shows minimal MSE as a function of λ. Note that the minimal point defines the value of optimal λ for the particular case. So, the procedure for determining λ_opt_ consists of finding the minimum point of the minimal MSE and λ graph.

The optimal number of steps is defined as the number of steps needed to reach a minimal MSE value. In Fig. 3E, we see that the optimal number of steps varies between different λ values. For example, if λ is fixed and close to λ_opt_ (2.5 for this particular case), the deconvolution process requires more steps to reach the minimal MSE.

In practice, the original image is unknown and we cannot use MSE as a measure of the quality of deconvolved images. So, appropriate stopping criterion that does not depend on the original image is needed. For this we used the evolution of λ_lsq_ during iteration (Fig. 3F). The general behaviour of λ_lsq_ during the deconvolution process can be described as follows. In the beginning, λ_lsq_ values are small, increase to the maximum after which the value stabilizes to a certain nonzero level. We notice that the points where MSE is minimal and where λ_lsq_ obtains a maximum value are correlated (Fig. 3A and 3F). Thus, the evolution of λ_lsq_ can be used as a stopping criterion.

**Fig. 5:**
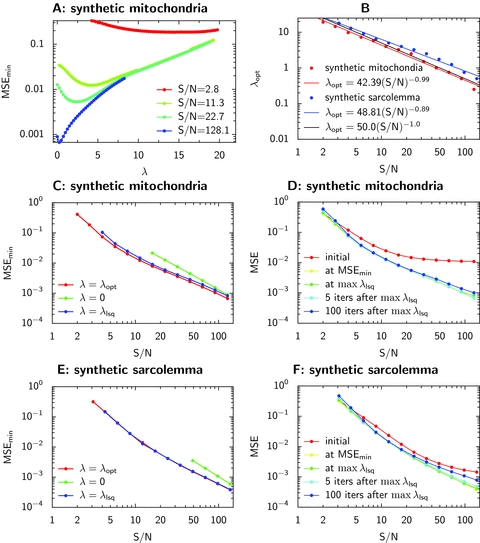
The analysis of deconvolution at different SNR. (A) Minimal MSE as a function of λ for various SNR values. Note that the optimal λ (defined as the minimum point of MSE_min_) is smaller for higher SNR. The original image is achieved only when noise is not present and the TV term is disabled (λ= 0). (B) The relations between SNR and optimal λ for two different types of image textures. The confocal images of mitochondria and sarcolemma in cardiac cells are used to obtain synthetics image with blocky- and honeycomb-like textures, respectively. Note the approximate exponential relations λ_opt_∼ 1/SNR, see text for details. (C) The relation between SNR and minimal MSE for disabled TV, estimated λ and optimal λ values, respectively. Restored image contains blocky-like textures. (D) MSE as a function of SNR and stopping iteration step. (E) Same as C, restored image contains honeycomb-like texture. (F) Same as D, restored image contains honeycomb-like texture.

Similar analysis was performed on test images with different SNR values as well as for honeycomb-like texture cases. Overall, there were 13 different test cases with various SNRs. From our simulations it is clear that for different SNR the value of λ_opt_ is not the same. In Fig. 5A where the MSE _min_ is displayed as a function of λ, we see that the minimum points of graphs are shifting towards zero as the SNR increases. Therefore, the value of λ_opt_ is decreasing with the increase of SNR. Note that when deconvolving images without noise (SNR =∞) and taking λ= 0, the original image is obtained (MSE → 0). By contrast, when deconvolving images with noise, the iteration process never converges to the original image. Furthermore, from the analysis of the values of λ_opt_ and SNR in Fig. 5B, we notice an inverse relation between λ_opt_ and SNR: 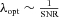. As a robust estimate, we suggest using λ= 50/SNR.

For microscope images we use λ_lsq_ because finding the optimal λ is a tedious and nontrivial process. By comparing the deconvolution results for synthetic images that use λ_lsq_, λ_opt_ and λ= 0 at different noise levels, we assessed the performance of the λ estimating formula. Figs 5C and E summarize the deconvolution results in terms of minimal MSE for different noise levels and λ selections. From the graphs we conclude that using optimal regularization always gives better results (smaller MSE), and generally, using regularization is necessary in the presence of noise. In addition, there exist a range of SNR values (5–100) where using λ_lsq_ gives the same order of magnitude for minimal MSE as using λ_opt_.

As it was suggested earlier, evolution of λ_lsq_ can be used as a stopping criterion. We suggest to stop the iteration process after five steps of obtaining λ_lsq_ maximum value. According to our simulations, such stopping criteria leads to deconvolved image with the resulting MSE close to minimal MSE (Figs 5D and F). In addition, long and converging iterations (100 iterations after λ_lsq_ maximum) cause MSE to diverse from the minimal MSE. This clearly shows that converging iteration does not guarantee more accurate results.

### Deconvolving microscope images

As an example, we applied RL algorithm with TV regularization to experimentally recorded images. First, confocal images of mitochondria and sarcolemma in rat cardiomyocytes were deconvolved. Secondly, to test the performance of the algorithm, we deconvolved confocal image with the punctated stain and a widefield image.

**Fig. 6:**
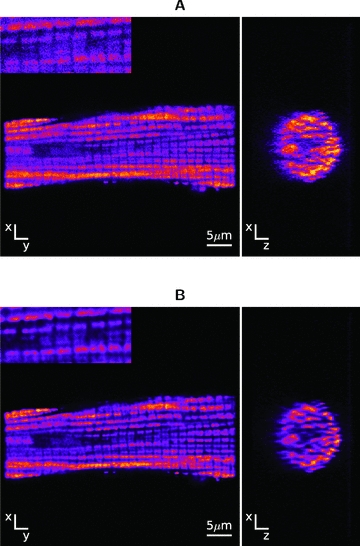
The image of mitochondria in rat cardiomyocyte before and after deconvolution algorithm was applied (voxel size 0.095 μm × 0.095 μm × 0.343 μm). The recorded confocal microscope image of rat cardiomyocyte mitochondria labelled with MitoTracker Green FM, note the blocky texture. (A) *xy* and *yz* cross-sections of the recorded image. At upper left corner, a zoomed region from the middle of *xy* cross-section is shown. (B) The seventh iteration of deconvolved recorded image A using estimated λ. Note the improvement in contrast. Noise is smoothed out and space between mitochondria has cleared.

In Fig. 6A, where the cell was labelled with MitoTracker Green FM, cross-sections *xy* and *yz* are displayed from the middle of cell. At upper left corner, an enlarged view from the middle part of the *xy* cross-section is shown. From the comparison of the recorded and deconvolved image using λ_lsq_, we can clearly see significant improvement in the quality of the deconvolved image (compare Figs 6A and B). For example, the noise is reduced considerably on the whole image and mitochondria can be more easily distinguished from each other. Note the significant contrast enhancement on *yz* plane.

On the basis of our analysis, we estimated that the optimal λ value for deconvolving the image of the cell with stained mitochondria (Fig. 6A) is 2.5. This estimation was made assuming that the relationship between the SNR and λ_opt_ (Fig. 5B) is valid for this recording as well. Comparing the result to one obtained using λ_lsq_, there are no visible differences (results not shown).

**Fig. 7:**
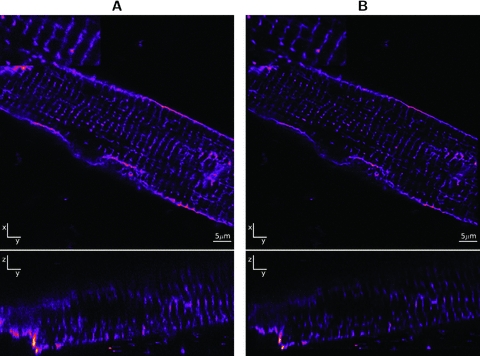
The image of sarcolemma in rat cardiomyocyte before and after deconvolution algorithm was applied (voxel size 0.063 μm × 0.063 μm × 0.387 μm). The recorded confocal microscope image of rat cardiomyocyte sarcolemma labelled with di-8-ANEPPS, note the honeycomb-like texture. (A) *xy* and *yz* cross-sections of recorded image. (B) The eighth iteration of deconvolved recorded image A with estimated λ. Note the improvement in contrast, noise is smoothed out, and *t*-tubules are more visible.

Fig. 7A shows a sarcolemma in rat cardiomyocyte labelled with di-8-ANEPPS. Lines seen in this image correspond to *t*-tubules. As in the previous example, two cross-sections and an enlarged view from the middle of the image are shown. From the comparison of recorded image and restored image (Figs 7A and B), we see an improvement in contrast and reduction of noise.

**Fig. 8:**
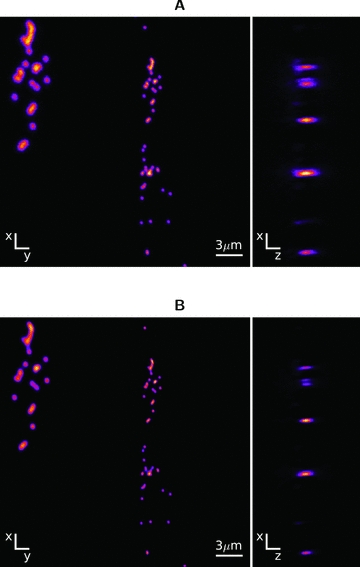
The image of microspheres cluster before and after deconvolution algorithm was applied (voxel size 0.054 μm × 0.054 μm × 0.188 μm). The recorded confocal microscope image of microspheres is also used for estimating PSFs, note punctated texture. (A) *xy* and *yz* cross-sections of the recorded image. At upper left corner, a zoomed region from the middle of *xy* cross-section is shown. (B) The eighth iteration of deconvolved recorded image A using estimated λ.

To test the performance of the algorithm, we deconvolved a confocal image of a cluster of microspheres. Such cluster is similar to punctated stain which can occur in live cells when imaging distribution of ryanodine receptors, for example. As it is shown in Fig. 8, experimental images can be successfully deconvolved for such texture as well.

**Fig. 9:**
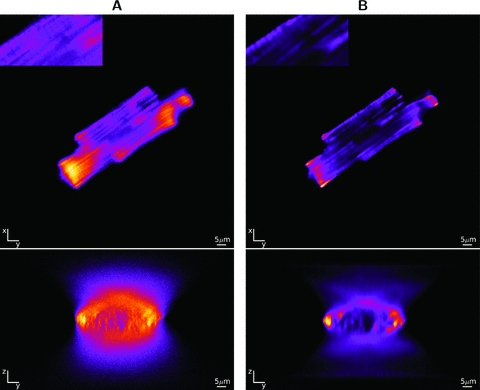
The image of mitochondria in rat cardiomyocyte before and after deconvolution algorithm was applied (voxel size 0.132 μm × 0.132 μm × 0.398 μm). The recorded widefield fluorescence microscope image (A) of rat cardiomyocyte mitochondria labelled with MitoTracker Green FM, note the blocky texture and extensive out-of-focus light. The image is improved by deconvolution (B) leading to the reduction of out-of-focus light and reducing noise. Note that deconvolution was not able to fully remove out-of-focus light.

Although the considered deconvolution algorithm is designed for confocal microscopy where Poisson noise is dominating, we have applied it to images of stained mitochondria of rat cardiomyocytes (Fig. 9A) acquired with a widefield fluorescence microscope. The deconvolution of such images reduces considerably out-of-focus light and reduces noise, as shown in Fig. 9B. However, further research is needed to improve the deconvolution result by taking into account other noise properties as well as gradients in the background field of widefield images.

## Discussion

In this work, we derived a formula to estimate the TV regularization parameter for regularized RL deconvolution algorithm and developed an open source software platform IOCBio Microscope where other deconvolution algorithms can be introduced easily. In addition, we illustrated that over a certain range of SNR, the estimated λ gives as good results as with the optimal regularization parameter. As a result, we propose a practical method to deconvolve confocal microscope images that uses estimated regularization parameter depending on the input image.

### Deconvolving synthetic images

We analysed the behaviour of the deconvolution algorithm on a synthetic image. Usually, synthetic images that are used in the analysis of deconvolution contain various geometrical shapes with different intensities. In this paper, we use synthetic images that are constructed from actual microscope images as described in the Materials and Methods section. Usage of such synthetic images gives us an opportunity to study the deconvolution algorithms under more realistic conditions.

The RL algorithm with TV regularization requires the selection of an appropriate regularization parameter value for each image that is being deconvolved. In practice, the selection of this parameter value is based on an educated guess. However, the analysis of synthetic images (Fig. 5A) shows that the errors can be an order of magnitude smaller with appropriate λ value than with arbitrary λ values. In addition, Fig. 5B shows that the optimal λ depends on the SNR exponentially: λ_opt_≈ 50/SNR.

Recently, methods for estimating the regularization parameter have been introduced to avoid the tedious work needed for finding the appropriate λ value. For this, Gilboa *et al.* (2003) use an adaptive variational scheme and Liao *et al.* (2009) make use of a generalized cross-validation technique. In this paper, we derived a formula Eq. (5) that is based on a least squares method and is related to findings of Gilboa *et al.* (2003). The usage of our formula gives as good results as with optimal λ value for a certain range of SNRs (Figs 5C–F).

### Estimated regularization parameter as stopping criteria

As a stopping criterion, several authors have used the relative change between two estimates (τ_1_) and the stopping point is determined when the relative change falls below a given threshold (van Kempen *et al.*, 1997; Dey *et al.*, 2004; Pankajakshan *et al.*, 2008). We could not use this approach for multiple reasons. First, we could not identify τ_1_ threshold value that would fit deconvolution processes with different TV regularization parameter values. Secondly, the value of τ_1_ was not always related to convergence of deconvolution process. Namely, we have shown (Figs 3A and B) that MSE can stabilize at relatively high values of τ_1_ in some cases and the opposite can be true as well (evolving MSE at small values of τ_1_). The discrepancy between stable MSE and relatively large τ_1_ was related to oscillations between several estimates obtained in successive deconvolution steps, as shown using τ_2_/τ_1_ relationship in the Results. Thus, according to our analysis, the use of τ_1_ for stopping deconvolution process would not lead to the best possible estimation of the ‘true’ image.

Our approach uses the evolution of λ_lsq_ that is computed from Eq. (5) as a stopping criterion – the deconvolution process is stopped after five iterations of λ_lsq_ has obtained its maximum value. The analysis of MSE confirms that the minimum point of MSE is well correlated with the point where λ_lsq_ obtains maximum value. By contrast, if the deconvolution process is prolonged, visually identifiable artefacts will be produced. This effect is well seen in Figs 5D and F where the results of long iterations (100 iterations after λ_lsq_ maximum has been obtained) have noticeably larger MSE compared to the results that are stopped after λ_lsq_ maximum point.

### Deconvolving confocal microscope images

Although the quality of deconvolution result strongly depends on the quality of the input data, the deconvolution can improve the quality of image, even if the data is greatly corrupted by noise.

However, to ensure a realistic result using iterative deconvolution algorithms, the process needs to be stopped before artefacts are created. For deconvolving microscope images, we used the RL algorithm with TV regularization using estimated λ. The optimal solution with noticeable improvements is achieved with a rather small number of iteration steps. However, prolonged iteration starts to produce artefacts. When using our λ estimation formula, the deconvolution process can be stopped at the right iteration step by monitoring the evolution of λ_lsq_. By contrast, when visually examining the estimates around λ_lsq_ maximum, they look equally acceptable (Fig. 4). Furthermore, when using this criterion with experimental data, we noted that at higher SNRs in the initial data, the optimal number of iteration steps is larger than for smaller SNRs. This is in accordance to the results obtained from the analysis of deconvolution of synthetic images.

The input for estimating the regularization parameter of the RL deconvolution algorithm is the peak SNR of a recorded image. With confocal microscopes that use photon counting detectors, the peak SNR can be directly estimated as a square of largest count value in image data. In practice, use of peak SNR can be problematic. First, staining artefacts leading to small cluster of bright pixels would determine the estimate of the ratio. Secondly, when non-photon counting confocal microscopes are used, the use of a square of largest count value in image data as an estimate of SNR is questionable. Indeed, confocal microscopes equipped with analogue light detectors record intensity in arbitrary units that depend on user settings of detector gain and offset. The both problems can be resolved if more general SNR estimate is used. For example, SNR estimate based on the variations of neighbouring voxels takes into account information from the whole image (de Monvel *et al.*, 2001). This would dampen the effect of small bright clusters in an image to SNR and, in addition, is applicable to images recorded using analogue detectors. How such estimate of SNR is related to the regularization parameter of the RL deconvolution algorithm is a subject of further studies.

In addition to difficulties in estimating SNR, confocal microscopes equipped with analogue detectors can have noise properties that are different from Poisson noise. However, that can be altered by user in practice by selecting lower gain of the detector. As it has been shown earlier, with lower gain settings, Poisson noise is dominating in images (Cho & Lockett, 2006). Furthermore, by averaging image during acquisition, user can improve general SNR (Conchello & Lichtman, 2005) leading to a better deconvolution of the image.

Another important element in deconvolution is the PSF which should be determined as accurately as possible to account for imperfections in the optical pathway. In this paper, we used PSFs obtained from the measurements of microspheres. Such PSFs account for imperfections such as asymmetry. Including asymmetry to computed PSF is not trivial because the source of the asymmetry is hard to determine. The accurate PSF is important because deconvolving with a incorrect PSF can result misleading conclusions (Cannell *et al.*, 2006). The quality of PSF plays a critical role in obtaining a high quality result, and to this end, we recommend using a measured PSF.

### IOCBio Microscope – a software for deconvolving microscope images

In this work, our contribution includes the development of an open source software package IOCBio Microscope that collects all necessary elements for deconvolution using the RL algorithm with TV variation regularization (Peterson, 2010a). This includes, reading microscope images of various formats (TIFF, RAW and LSM files), estimating PSF from the measurements of microspheres, deconvolving images with different algorithm options, etc. The software is implemented in Python which has proven itself as an excellent prototype language for testing algorithms. The computationally expensive parts are implemented in C and FFTW library is used with multiple threads to reduce CPU time for deconvolution considerably. For example, deconvolving an image stack of size 32 × 512 × 512 with 100 iterations takes about 10 min on a standard desktop computer. We anticipate that this deconvolution software package becomes a platform for testing different deconvolution algorithms. Furthermore, the software has reasonable graphical user interface that makes it easy to use for enhancing microscope images. The software can be run in a computer cluster environment such as Sun Grid Engine to parallelize deconvolution tasks.

In future, we plan to implement Poisson noise removal algorithm by Le *et al.* (2007) that can be applied to estimated PSF as well as to the first estimate of deconvolution process. Application of such a noise removal can improve the efficiency of deconvolution algorithms even further. In addition, we plan to add interfaces to existing C/C++ deconvolution libraries such as Clarity and Deconv so that these algorithms could be used from the Python based IOCBio Microscope package. The availability of semi-automatic wrapper generation tools like SWIG (Beazley, 2003) and F2PY (Peterson, 2009) as well as the standard Python ctypes module makes wrapping C, C++ or Fortran software libraries to Python-based platform particularly easy. See Peterson (2010c) for an example of wrapping the Deconv library to Python.

Although this paper deals with confocal microscopy and deconvolving its images, the IOCBio Microscope software has extensions to apply deconvolution algorithms to the images of widefield microscopy as well. In particular, the algorithm for estimating PSF from the measurements of microspheres is adapted to deal with specific properties of widefield microscopy recordings such as relatively high level and nonuniform background field, different noise model, etc. The software can be used for testing deconvolution algorithms on recordings of widefield microscopy, similarly to what we have done in this paper for recordings of confocal microscopy. However, as seen in Fig. 9, more work is required to improve further the deconvolution results of widefield images by using the RL algorithm. It must take into account more appropriate image formation model for a widefield microscope that have other noise properties and more dominant background field gradients than in confocal microscope.

## Conclusions

To conclude, we have developed an open source software package IOCBio Microscope that can be used for deconvolving images in practice. The developed software package can be also used as a platform for testing new deconvolution algorithms. We have derived a formula to estimate the TV regularization parameter for regularized RL deconvolution algorithm and shown that over a certain range of noise levels the estimated regularization parameter gives as good results as the optimal regularization parameter. Inverse relation between the optimal TV regularization parameter and image SNR is shown and taken into account in the regularization estimation. In addition, new stopping criterion for deconvolution process has been proposed.
